# Changes in Choline Metabolites and Ceramides in Response to a DASH-Style Diet in Older Adults

**DOI:** 10.3390/nu15173687

**Published:** 2023-08-23

**Authors:** Brianna N. Tate, Gary P. Van Guilder, Marwa Aly, Lisa A. Spence, M. Elena Diaz-Rubio, Henry H. Le, Elizabeth L. Johnson, Joseph W. McFadden, Cydne A. Perry

**Affiliations:** 1 Department of Animal Science, Cornell University, Ithaca, NY 14853, USA; btate@som.umaryland.edu (B.N.T.); jwm43@cornell.edu (J.W.M.); 2 High Altitude Exercise Physiology Department, Western Colorado University, Gunnison, CO 81231, USA; gvanguilder@western.edu; 3 Department of Applied Health Science, Indiana University School of Public Health, Bloomington, IN 47405, USA; mealy@iu.edu (M.A.); lisspenc@iu.edu (L.A.S.); 4 Proteomic and Metabolomics Facility, Cornell University, Ithaca, NY 14853, USA; m_elenadr@hotmail.com; 5 Division of Nutritional Sciences, Cornell University, Ithaca, NY 14853, USA; hhl43@cornell.edu (H.H.L.); elj54@cornell.edu (E.L.J.)

**Keywords:** DASH diet, choline, older adults, TMAO, ceramide, lysophostidylcholine

## Abstract

This feeding trial evaluated the impact of the Dietary Approaches to Stop Hypertension diet on changes in plasma choline, choline metabolites, and ceramides in obese older adults; 28 adults consumed 3oz (n = 15) or 6oz (n = 13) of beef within a standardized DASH diet for 12 weeks. Plasma choline, betaine, methionine, dimethylglycine (DMG), phosphatidylcholine (PC), lysophosphotidylcholine (LPC), sphingomyelin, trimethylamine-N-oxide (TMAO), L-carnitine, ceramide, and triglycerides were measured in fasted blood samples. Plasma LPC, sphingomyelin, and ceramide species were also quantified. In response to the study diet, with beef intake groups combined, plasma choline decreased by 9.6% (*p* = 0.012); DMG decreased by 10% (*p* = 0.042); PC decreased by 51% (*p* < 0.001); total LPC increased by 281% (*p* < 0.001); TMAO increased by 26.5% (*p* < 0.001); total ceramide decreased by 22.1% (*p* < 0.001); and triglycerides decreased by 18% (*p* = 0.021). All 20 LPC species measured increased (*p* < 0.01) with LPC 16:0 having the greatest response. Sphingomyelin 16:0, 18:0, and 18:1 increased (all *p* < 0.001) by 10.4%, 22.5%, and 24%, respectively. In contrast, we observed that sphingomyelin 24:0 significantly decreased by 10%. Ceramide 22:0 and 24:0 decreased by 27.6% and 10.9% (*p* < 0.001), respectively, and ceramide 24:1 increased by 36.8% (*p* = 0.013). Changes in choline and choline metabolites were in association with anthropometric and cardiometabolic outcomes. These findings show the impact of the DASH diet on choline metabolism in older adults and demonstrate the influence of diet to modify circulating LPC, sphingomyelin, and ceramide species.

## 1. Introduction

Aging presents with body composition changes that include declines in muscle health with simultaneous increases in body fat. These changes increase the risk for cardiometabolic abnormalities such as insulin resistance, hyperglycemia, visceral adiposity, dyslipidemia, and hypertension [1,2,3], resulting in the development of obesity, type-2 diabetes mellitus (T2DM), and cardiovascular disease (CVD). Older adults are disproportionately affected by cardiometabolic abnormalities as they are more likely to experience co-existing risk factors. Diet quality influences the onset of cardiometabolic disease and dietary patterns are vital to the quality of life and survival of older adults [4,5,6]. Currently, 41.5% of older adults in the United States (US) are obese [7] and 80% have two or more risk factors related to cardiometabolic disease [8]. Furthermore, the 2020 US census reports that there are 55.8 million older adults in the US and, by the year 2060, the population is projected to increase to 95 million [9]. With current medical costs related to cardiometabolic health estimated to be USD 50.4 billion and costs being highest among older adult men [10], implementation of effective diet therapies that improve cardiometabolic health, enhance the quality of life in older adults, and reduce economic burdens are greatly needed.

Choline, a micronutrient grouped with the family of B-complex vitamins, has received considerable attention due to its role in one-carbon metabolism, neurotransmission, membrane synthesis, and lipid transport [11,12,13]. Choline metabolism is divided into multiple pathways resulting in the synthesis of betaine, phosphatidylcholine (PC), lysophosphotidylcholine (LPC), sphingomyelin, and trimethylamine-N-oxide (TMAO) [14]. Dimethylglycine (DMG) is formed through betaine metabolism by the action of betaine-homocysteine methyltransferase, which takes place within the choline metabolic pathway [15,16]. DMG and betaine are used in the remethylation of homocysteine to form the amino acid methionine [17]. Collectively, these metabolites have a broad range of physiological functions across all stages of the life cycle and are implicated in the pathogenesis of obesity, T2DM, and CVD. Most human trials, however, reporting on the relationship between choline metabolites and metabolic health are not interpreted within the context of diet, dietary pattern, or nutritional status, which may lead to conflicting outcomes and difficulty interpreting results. For instance, studies report that LPCs have anti-inflammatory and pro-inflammatory properties and are elevated and reduced in obesity and T2DM [18,19,20,21,22]. Studies also show that high plasma sphingomyelins are associated with incident of and reduced risk of heart failure and death [23,24,25]. It is known that diet directly influences metabolic health in humans, however, the impact of diet on the profile of choline metabolites in relation to metabolic health is largely unexplored, particularly in vulnerable populations such as older adults.

Ceramide is a sphingolipid that can be synthesized from the hydrolysis of sphingomyelin [26]. Ceramide interferes with insulin signaling and promotes uncontrolled adipose tissue lipolysis and inflammation [27,28]. Circulating ceramide concentrations are elevated in older adults and individuals with metabolic syndrome and T2DM [29,30,31,32]. Whether diet has the ability to decrease ceramide levels in association with increased metabolic health is relatively unknown. A low-fat diet based upon the Dietary Guidelines for Americans was shown to decrease circulating ceramide and inflammation in overweight adults [32], indicating that diet may be an approach to reduce circulating ceramide with resulting improvements in health in humans.

The Dietary Approaches to Stop Hypertension (DASH) dietary pattern is a high-quality therapeutic diet known to improve health in vulnerable populations, resulting in improved cardiovascular and cognitive health and reductions in metabolic diseases, including T2DM, metabolic syndrome, and non-alcoholic fatty liver disease [33,34,35,36,37]. In a 12-week controlled feeding trial, we reported improved body composition and cardiometabolic health in response to a DASH-like diet in obese older adults described by reduced body fat, improved muscle health, decreased inflammation, and enhanced insulin sensitivity [38,39,40]. Extending the scope of these findings and given that choline metabolites and ceramide may play role in human metabolic health, we sought the following: (i) to evaluate the changes in plasma choline, choline metabolites, and ceramide in response to the DASH diet in older adults; and (ii) to assess the impact of the DASH diet on changes in individual LPC, sphingomyelin, and ceramide species. Considering that the relationship between diet and choline metabolism in older adults is relatively unknown, this study aims to contribute to this unexplored gap in knowledge.

## 2. Materials and Methods

### 2.1. Study Participants

Characteristics and recruitment of participants have been previously reported [38]. Briefly, adults aged 65–84 years were recruited from the Brookings, South Dakota area from June 2017 to August 2018. Prior to enrollment, participants completed a questionnaire that included date of birth and medication, vitamin, drug, and alcohol use. Inclusion criteria were: age, upward mobile ability, and compliance with the study protocol. A full characterization of body composition measures and cardiometabolic outcomes has been previously published [38,39]. This study was registered with ClinicalTrials.gov, identifier: NCT04127240.

### 2.2. Study Design, Body Composition Measures, and Blood Sample Collection

A human controlled feeding trial with a parallel design was performed in which females (n = 17) and males (n = 11) aged 65+ years were randomized to consume either 3 oz (85 g; n = 15) or 6oz (170.1 g; n = 13) of lean fresh beef within a standardized DASH diet. As previously described, Nutritionist Pro™ software was used to create the study diet. The nutrient composition was based upon the 2015–2020 dietary guidelines for daily caloric intake for older sedentary adults and the DASH eating plan using the National Heart, Lung, and Blood Institute, National Institutes of Health [41,42]. The macronutrient profile of the study diet was previously published [43]. Equal amounts of beef were distributed amongst three major meals: breakfast, lunch, and dinner. Participants were required to consume at least one meal per day in the food laboratory at South Dakota State University Monday through Friday. All other meals, snacks, and beverages were provided as takeaways. A multivitamin supplement for seniors (Hy-Vee Health Market Senior Multivitamin & Multimineral Supplement, Hy-Vee Health Market, West Des Moines, IA, USA) was provided daily. Investigators had consistent contact with participants throughout the study and participants were required to verify the consumption of each food item by completing a daily checklist provided by investigators.

Body composition and cardiometabolic methods have been previously reported [38,39]. Briefly, total body weight, %body fat, and SMM were measured by bioelectrical impedance (InBody 270, InBody, Cerritos, CA, USA); BMI was calculated using weight (kg) divided by height (m); grip strength was quantified using a hand-held dynamometer (Smedley III analog); the Dimension Xpand plus integrated chemistry automated analyzer (Siemens Healthineers, Malvern, PA, USA) was used to measure total cholesterol, LDL-C, and glucose; the Immulite 2000 automated immunoassay system (Siemens Healthineers, Malvern, PA, USA) was used to measure CRP and insulin; Meso Scale Discovery (Meso Scale Diagnostics, LLC, Rockville, MD, USA) measured IL-8; HOMA-IR was calculated using: fasting plasma glucose (mmol/L) times fasting serum insulin (μIU/mL) divided by 22.5. Myostatin was measured using the human quantikine myostatin immunoassay solid phase enzyme-linked immunosorbent assay (ELISA; R&D systems, Minneapolis, MN, USA).

Participants provided fasted blood samples at weeks 0, 3, 6, 9, and 12. Due to limited resources for analysis, for this study measures were performed at weeks 0, 6, and 12. The fasted blood samples were collected into 10 mL serum separator clot activator tubes (SST Vacutainer; Pulmolab, Northridge, CA, USA) and EDTA-coated tubes (Pulmolab) by a trained phlebotomist. Blood for serum collection was kept at room temperature, allowed to clot, and centrifuged at 650× *g* for 15 min at room temperature. The 10 mL EDTA-coated tubes containing blood were put on ice immediately after blood collection and centrifuged within 90 min at 1055× *g* for 15 min at 4 °C. All samples were aliquoted into 1.8 mL cryostat vials and stored at −80 °C.

### 2.3. Choline, Betaine, Trimethylamine N-Oxide, and L-Carnitine Quantification

We utilized LC-MS/MS to quantify choline and betaine as previously described [44] with modifications to include measurements of TMAO. Briefly, 100 µL of 0.1% formic acid in acetonitrile and 5 µL of internal standard mix were added to 50 µL of plasma. Internal standard mix contained choline D13 (CDN Isotopes, Pointe-Claire, QC, Canada), betaine D3 (CDN Isotopes), TMAO D9 (Cambridge Isotopes, Tewksbury, MA, USA). After vortexing and centrifugation, 5µL of clear supernatant was injected into a Syncronis Silica 150 × 2.1, 5u with matching guard column (Thermo Scientific, Waltham, MA, USA). Metabolites were separated under isocratic conditions using 19% of 15 mM ammonium formate with 0.1% formic acid and 81% acetonitrile with flow of 0.5 mL/min. Calibration curves were generated by serial dilutions of unlabeled metabolites in water with the addition of a comparable amount of internal standard mix (i.e., same concentrations as samples). Positive ion SRM transitions were *m*/*z* 76 to 58 and *m*/*z* 85 to 66 for TMAO and TMAO D9, respectively. L-carnitine was quantified using a commercial colorimetric kit (Sigma-Aldrich, St. Louis, MO, USA).

### 2.4. Ceramide Quantification

Plasma samples (700 µL) were frozen over liquid nitrogen and lyophilized to dryness. HPLC-grade methanol (1.4 mL) was added to the dried material and the mixture was sonicated for 3 min (on/off pulse cycles of 3 s on, 2 s off, at power 100%) using a Qsonica Ultrasonic Processor (Model Q700; Qsonica, Newtown, CT, USA) with a water bath cup horn adaptor (Model 431C2) and the water bath maintained at room temperature. Samples were placed on an end-over-end rotator, and extraction continued for 24 h, then centrifuged at 18,000× *g* for 30 min at 4 °C. The clarified extract was transferred to a fresh centrifuge tube and dried with a Thermo Scientific Savant SpeedVac SPD130DLX (Thermo Scientific, Waltham, MA, USA). The dried material was resuspended in 200 μL HPLC-grade methanol, briefly sonicated, and centrifuged as before. The concentrated extract was then transferred to an HPLC vial with a 300 μL glass insert and stored at 4 °C until further analysis.

Ceramide analyses were performed using a Thermo Scientific Vanquish Horizon UHPLC System coupled with a Thermo Scientific TSQ Quantis Triple Quadrupole mass spectrometer equipped with a HESI ion source. Mobile phase A was 78.6% water, 20% acetonitrile, and 0.4% formic acid (*v*/*v*). Mobile phase B was 47.8% methanol, 47.8% acetonitrile, 4% chloroform, and 0.4% formic acid. Briefly, 5 µL of extract was injected and separated on a mobile phase gradient with an Agilent Technologies InfinityLab Poroshell 120 EC-C18 column (50 mm × 2.1 mm, particle size 2.7 μm, part number: 699775-902; Agilent Technologies, Santa Clara, CA, USA) maintained at 50 °C. All solvents were purchased as HPLC-grade from Fisher Scientific. A/B gradient started at 10% B for 1 min after injection and increased linearly to 100% B at 9 min and held at 100% B for 10 min, using a flow rate 0.6 mL/min. Full Scan Q1 mass spectrometer parameters: spray voltage 4.8 kV in positive mode, ion transfer tube temperature 275 °C, vaporizer temperature 250 °C; sheath, auxiliary, and spare gas 53.2, 16.3, and 3.3, respectively. The mass spectrometer was calibrated with the Pierce Triple Quadrupole Calibration Solution Extended Mass Range solution. Samples were analyzed in positive mode with *m*/*z* range 150 to 1000, and all metabolites were verified with synthetic standards purchased from Avanti Polar Lipids (Avanti Polar Lipids, Alabaster, AL, USA).

### 2.5. Lysophosphatidylcholine Quantification

Stock lipid standards were prepared by dissolving them in dichloromethane (DCM)/methanol (MeOH; 2:1 *v*/*v*) at concentration ranging from 1 to 5 mg/mL and were stored at −20 °C. The final internal standard mixture spiked into each sample before extraction consisted of 25 μg/mL of TG 15:0/15:0/15:0 (Sigma-Aldrich), phosphatidylglycerol 14:0/14:0 (Avanti Polar Lipids), phosphatidylserine 16:0/16:0, ceramide 18:1/12:0 (Avanti Polar Lipids), cholesteryl ester 17:0 (Avanti Polar Lipids), and 5 μg/mL LPC 18:D17 (Avanti Polar Lipids) and phosphatidylcholine d18:1D7/15:0 (Avanti Polar Lipids).

Thirty microliters of internal standard mixture were added to 30 μL of sample followed by 190 μL of MeOH. Samples were then vortexed for 20 s. Next, 380 μL of DCM was added, the sample was vortexed for 20 s, and 120 μL of water was added to induce phase separation. The samples were then vortexed for 10 s and allowed to equilibrate at room temperature for 10 min before centrifugation at 8000× *g* for 10 min at 10 °C. A total of 370 μL of the lower lipid-rich DCM layer was then collected and the solvent was evaporated to dryness under vacuum. Samples were reconstituted in 200 μL of acetonitrile (ACN)/2-propanol (IPA)/water (65:30:5 *v*/*v*/*v*) before chromatographic separation.

Chromatographic separation was performed on a Vanquish UHPLC system with an Accucore C30, 2.6 μm column (2.1 mm id × 150 mm) coupled to a Q Exactive™ Hybrid Quadrupole-Orbitrap High Resolution Mass Spectrometer (Thermo Scientific). The mobile phase consisted of (solution A) 60% ACN, 40% water, 10 mM ammonium formate with 0.1% formic acid and (solution B) 90% IPA, 10% ACN, 10 mM ammonium formate with 0.1% formic acid. The gradient was as follows: 0 to 1.5 min, 32% solvent B; 1.5 to 4 min, 32 to 45% solvent B; 4 to 5 min, 45 to 52% solvent B; 5 to 8 min 52 to 58% solvent B; 8 to 11 min, 58 to 66% solvent B; 11 to 14 min, 66 to 70% solvent B; 14 to 18 min, 70 to 75% solvent B; 18 to 21 min, 75 to 97% solvent B; 21 to 25 min, isocratic 97% solvent B; 25 to 25.1 min, 97 to 32% solvent B; followed by 4 min of re-equilibration of the column before the next run. The flow rate was 260 μL/min. All of the samples were analyzed by electrospray ionization in data-dependent MS/MS mode.

Nitrogen was used as the nebulizing gas, with sheath, auxiliary, and sweep gas flows set at 50, 5, and 1 arbitrary units, respectively. Other conditions included the following: resolution, 120,000 full width at half maximum; automatic gain control target, 3 × 10^6^ ions; maximum injection time, 100 ms; scan range, 67 to 1000 m/z; spray voltage, 3 kV; and capillary temperature, 320 °C. Data-dependent MS/MS spectra were generated using the following conditions: resolution, 15,000 full width at half maximum; automatic gain control target, 1 × 10^5^ ions; maximum injection time, 50 ms; isolation window, 0.4 m/z; and stepped normalized collision energies, 25 and 35. The sequence of injections was randomized to avoid possible bias.

Acquired data were processed using LipidSearch™ software version 4.3 (Thermo Scientific) with the following workflow: first, the individual data files were searched for product ion MS/MS spectra of lipid precursor ions. MS/MS fragment ions were predicted for all precursor adduct ions measured within ±5 ppm. The product ions that matched the predicted fragment ions within a ±5 ppm mass tolerance were used to calculate a match-score, and those candidates providing the highest-quality match were determined. Next, the search results from the individual positive or negative ion files from each sample group were aligned within a retention time window (±0.1 min) and the data were merged for each annotated lipid. The annotated lipids were then filtered to reduce false positives.

### 2.6. Statistical Analyses

Data were tested for assumptions of normality and spread using the Shapiro–Wilk test and normal distribution curves. Data that did not meet assumptions of normality were analyzed with non-parametric tests and reported as the median with interquartile range. Data were pooled by sex and by beef intake group and also displayed separately. Independent samples *t*-tests were performed to determine differences at baseline and week 12 by beef intake group and by sex. Levene’s test for equality of variances was performed to assess the homogeneity of variance assumption for all independent samples *t*-tests. When the assumption of equality of variances was not met, data are reported with the adjusted *p*-value. Mixed design ANOVA was used to determine changes in choline, choline metabolites, ceramide, and triglycerides in response to the 12-week intervention. When indicated by a significant time effect, the Bonferroni adjustment for multiple comparisons was used to determine pairwise differences at specific time points. To adjust for the influence of changes in potential confounding variables across the intervention, we repeated the mixed design ANOVA by including age, sex, BMI, glucose, and insulin as covariates. Concentrations of LPC, sphingomyelin, and ceramide species at baseline and week 12 by beef intake group were analyzed by an independent samples *t*-test. Mixed design ANOVA was used to determine changes in LPC, sphingomyelin, and ceramide species in response to the diet intervention. Friedman’s non-parametric test for repeated measures data and the Mann–Whitney U test for between-group comparisons were performed for data that did not meet assumptions of normality. Relations between the change from baseline in plasma choline, betaine, PC, L-carnitine, TMAO, sphingomyelins, ceramides, and LPCs and between changes in body composition, cardiometabolic, and inflammatory markers were examined by Pearson’s correlation coefficient. Statistical significance was set at *p* < 0.05. Data are presented as means (SD) and were analyzed with SPSS version 24 (IBM Inc., Armonk, NY, USA).

## 3. Results

### 3.1. Study Diet

Estimated choline, choline forms, L-carnitine, and methionine content of the study diet separated by beef group are shown in Table 1. Dietary choline and betaine content were calculated based on the 2022 United States Department of Agriculture (USDA) FoodCentral database estimations [45]. Each food and beverage item provided by the study diet was searched in the USDA FoodCentral database for choline and betaine content. When data were available for choline and betaine in mg/100 g food, this number was used to calculate the choline and betaine in mg per gram of food consumed in the study diet. The total choline (mg) and total betaine (mg) delivered by the 7-day cycle diet were divided by 7 to obtain an average daily consumption of the constituents. The total daily contributions of choline and betaine are reported. Participants randomized to the 3 oz beef group consumed an estimated 257 mg per day of choline and 28 mg per day of betaine. Participants in the 6 oz beef group consumed an estimated 336 mg per day of choline and 35 mg per day of betaine.

Given that the 2022 USDA database does not include choline forms, glycerophosphocholine (GPC), phosphocholine, PC, and sphingomyelin were calculated using the 2008 USDA Database for the Choline Content of Common Foods [46]. Each choline form was available in mg/100 g of food and this number was used to calculate the choline forms in mg per gram of food consumed. The total for each choline form provided by the 7-day cycle menu was divided by 7 to calculate an average daily consumption amount of each constituent. Participants randomized to the 3 oz beef group consumed an estimated 51 mg of GPC, 13 mg of phosphocholine, 79 mg of PC, and 14 mg of sphingomyelin. Participants randomized to the 6 oz beef group consumed an estimated 53 mg of GPC, 13 mg of phosphocholine, 85 mg of PC, and 21 mg of sphingomyelin.

L-carnitine is not included in the USDA database, thus the National Institutes of Health Office of Dietary Supplements Carnitine Fact Sheet for Health Professionals was used to calculate dietary L-carnitine in the beef provided by the study diet [47]. The Fact Sheet provides L-carnitine content of selected foods in mg per serving and lists 3 oz of beef as a food item. Using the Fact Sheet, participants randomized to the 3 oz beef group consumed an estimated average of 70 mg of L-carnitine each day and participants in the 6 oz beef group consumed an estimated average of 140 mg of L-carnitine daily.

Nutritionist Pro™ software was used to estimate dietary methionine content of the diet. Each food item from the study was put into the software and Nutritionist Pro™ calculated the amount of methionine consumed. Participants in the 3 oz group received 1350 mg of methionine and the 6 oz group received 1957 mg of methionine.

### 3.2. Baseline (Week 0) Characteristics of Study Participants

Participants (n = 28) 65–84 years of age completed the 12-week diet intervention and were included in the final analysis. As previously reported, all participants presented with the following at baseline: total body weight: 91.2 kg; BMI: 32 kg/m^2^; SMM: 31.4 kg; %body fat: 37.2%; grip strength: 62.6 kg; myostatin: 3.4 ng/mL; total cholesterol: 180.9 mg/dL; LDL-C: 104.5 mg/dL; glucose: 108.5 mg/dL; insulin: 14.1 μU/mL; HOMA-IR: 4.0; CRP: 2.8 mg/L; IL-8: 6.3 pg/mL [38,39,40]

Baseline characteristics separated by beef group are presented in Table 2. Participants randomized to the 3 oz beef group at baseline had higher (*p* = 0.050) plasma total LPC (30.7 μg/mL) compared to participants randomized to the 6 oz beef group (27.9 μg/mL). At baseline, participants randomized to the 3 oz beef group had higher (*p* = 0.023) plasma triglycerides (224 mg/dL) compared to participants randomized to the 6 oz beef group (155 mg/dL). There were no other statistically significant differences (*p* > 0.05) between the 3 oz and 6 oz beef groups at baseline.

Baseline characteristics separated by sex are presented in Table 3. Males had higher (*p* = 0.042) baseline choline levels (12.72 nM/mL) compared to females (10.68 nM/mL). Males had higher (*p* = 0.003) plasma methionine (37.51 μM/mL) compared to females (30.51 μM/mL). Plasma DMG was higher (*p* = 0.001) in males (3.13 μM/mL) compared to females (2.23 μM/mL). Females had higher (*p* = 0.050) sphingomyelin concentrations (141.5 μM/mL) compared to males (124.7 μM/mL) at baseline. There were no other statistically significant differences (*p* > 0.05) between males and females at baseline.

### 3.3. Week 12 Outcomes in Response to the Intervention

Week 12 results separated by beef group are presented in Table 2. At week 12, participants who consumed 6 oz of beef had higher (*p* = 0.033) circulating TMAO concentrations (5.2 nM/mL) compared to participants who consumed 3 oz of beef (4.6 nM/mL). Participants who consumed 6 oz of beef had higher (*p* = 0.003) plasma triglycerides (167 mg/dL) compared to participants who consumed 3 oz of beef (149 mg/dL) at week 12. There were no other statistically significant differences (*p* > 0.05) between the 3 oz and 6 oz beef groups at week 12.

Week 12 outcomes separated by sex are presented in Table 3. Males had higher (*p* = 0.040) week 12 betaine levels (41.4 nM/mL) compared to females (31.5 nM/mL). DMG was higher (*p* < 0.001) in males (2.96 μM/mL) compared to females (1.95 μM/mL). Males had higher (*p* = 0.003) triglyceride concentrations (170 mg/dL) compared to females (148 mg/dL) at week 12. There were no other statistically significant differences (*p* > 0.05) between males and females at week 12.

### 3.4. Changes in Plasma Choline, Choline Metabolites, Total Ceramide, and Triglycerides in Response to the Study Diet

Plasma choline, betaine, methionine, DMG, PC, total LPC, total sphingomyelin, TMAO, L-carnitine, total ceramide, and triglyceride responses to the diet intervention are shown in Table 4. Throughout the 12-week intervention period, responses to the study diet were detected for choline (*p* = 0.012), DMG (*p* = 0.042), total PC (*p* < 0.001), total LPC (*p* < 0.001), TMAO (*p* < 0.001), total ceramide (*p* < 0.001), and triglycerides (*p* = 0.021). These responses remained after including covariates in the analysis. In response, choline decreased by 9.6% from week 0 (11.5 nM/mL) to week 12 (10.4 nM/mL); DMG decreased by 13% from week 0 (median: 2.62 μM/mL) to week 12 (median: 2.27 μM/mL); total PC decreased by 51% from week 0 (1384.3 μg/mL) to week 12 (679.5 μg/mL); total LPC increased by 281% from week 0 (29.4 μg/mL) to week 12 (112 μg/mL); TMAO increased by 26.5% from week 0 (4.9 nMol/mL) to week 12 (6.2 nMol/mL); total ceramide decreased by 22.1% from week 0 (10.2 μM/mL) to week 6 (8.1 μM/mL); and triglycerides decreased by 18.2% from week 0 (192 mg/dL) to week 12 (157 mg/dL). There was no significant interaction (*p* = 0.863) between beef intake and total ceramide. However, we did observe a significant beef intake interaction for total LPC (*p* = 0.008) and TMAO (*p* = 0.01). The 3 oz beef group showed a higher total LPC at week 6 compared with the 6 oz beef group. The 6 oz beef group had higher TMAO at week 6 and week 12 compared with the 3 oz beef group. No other interactions were observed.

### 3.5. Changes in LPC, Sphingomyelin, and Ceramide Species in Response to the Diet Intervention

Baseline characteristics of LPC, sphingomyelin, and ceramide species separated by beef group are presented in Table 5. Baseline LPC species 18:0, 18:1, and 18:2 were higher (*p* < 0.05) in the 3 oz beef group compared with the 6 oz group. There were no other statistically significant differences (*p* > 0.05) among the LPC, sphingomyelin, and ceramide species between the 3 oz and 6 oz beef groups at baseline.

Week 12 results of LPC, sphingomyelin, and ceramide species separated by beef group are presented in Table 5. Participants who consumed 3 oz of beef had higher (*p* = 0.037) LPC 16:0e (0.53 μg/mL) compared to participants who consumed 6 oz of beef (0.51 μg/mL). At week 12, participants who consumed 6 oz of beef had higher (*p* = 0.033) LPC 26:0 (0.08 μg/mL) compared to participants who consumed 3 oz of beef (0.07 μg/mL). There were no other statistically significant differences (*p* > 0.05) among the LPC, sphingomyelin, and ceramide species between the 3 oz and 6 oz beef groups at week 12.

Changes in plasma LPC, sphingomyelin, and ceramide species in response to the diet intervention are presented in Table 5. Throughout the 12-week intervention period, all 20 LPC species measured increased (*p* < 0.01) from baseline to week 12. In response to the study diet, sphingomyelin 16:0 increased by 10.4% (*p* < 0.001) from baseline (45.3 μM/mL) to week 12 (50.0 μM/mL). Sphingomyelin 18:0 increased by 22.5% (*p* < 0.001) from baseline (11.1 μM/mL) to week 12 (13.6 μM/mL). Sphingomyelin 18:1 also increased ~24% (*p* < 0.001) from baseline (4.8 μM/mL) to week 12 (6.0 μM/mL). Sphingomyelin 24:0 decreased by 10% (*p* < 0.001) from baseline (34.1 μM/mL) to week 12 (30.8 μM/mL). Throughout the 12-week intervention period, ceramide 22:0 decreased by 26.2% (*p* < 0.001) from baseline (median: 1.22 μM) to week 12 (median: 0.90 μM); ceramide 24:0 decreased by 11.2% (*p* = 0.002) from baseline (median: 7.60 μM) to week 12 (median: 6.75 μM); and ceramide 24:1 increased by 37.5% (*p* = 0.054) from baseline (median: 0.56 μM) to week 12 (median: 0.77 μM).

### 3.6. Changes in Choline, Betaine, L-Carnitine, TMAO, Sphingomyelins, Ceramides, and LPCs in Correlation with Changes in Body Composition, Cardiometabolic, and Inflammatory Markers

Changes in choline, betaine, LPCs, sphingomyelins, TMAO, L-carnitine, and ceramides in association with body composition, cardiometabolic, and inflammatory markers are shown in Table S1. The change in choline (r = −0.44; *p* = 0.026) and betaine (r =−0.49; *p* = 0.012) was correlated with %body fat. The change in L-carnitine was correlated with glucose (r =−0.49; *p* = 0.011) and IL-8 (r = 0.47; *p* = 0.014). The change in TMAO was correlated with insulin (r = 0.51; *p* = 0.007) and HOMA-IR (r = 0.50; *p* = 0.01).

The change in sphingomeyelin 24:0 was correlated with total cholesterol (r = −0.42; *p* = 0.049), LDL-C (r = −0.49; *p* = 0.02), glucose (r= −0.63; *p* = 0.002), and IL-8 (r = 0.74; *p* = 0.0001). The change in sphingomeyelin 24:1 was correlated with total body weight (r = −0.61; *p* = 0.003), BMI (r = −0.61; *p* = 0.003), SMM (r = −0.47; *p* = 0.045), and grip strength (r = 0.51; *p* = 0.016). The change in ceramide 22:0 was correlated with total cholesterol (r = 0.44; *p* = 0.045).

Several LPC species were correlated with anthropometric and biomarkers of cardiometabolic health and inflammation (Table S1). The change in LPC 16:0 was correlated with myostatin (r = 0.48; *p* = 0.016). The change in LPC 16:1 (r = 0.52; *p* = 0.008) and 18:0 (r= 0.42; *p* = 0.039) was correlated with glucose. The change in LPC 18:1 (r = 0.42; *p* = 0.035) and 20:1 (r = 0.55; *p* = 0.005) was correlated with triglycerides. The change in LPC 18:2 was correlated with %body fat (r = 0.41; *p* = 0.04). The change in LPC 20:4 was correlated with total body weight (r = 0.39; *p* = 0.05), BMI (r = −0.39; *p* = 0.05), myostatin (r = −0.55; *p* = 0.004), total cholesterol (r = −0.43; *p* = 0.032), and LDL-C (r = −0.40; *p* = 0.046).

Numerous LPC species were correlated with the inflammatory markers CRP and IL-8. The change in LPC 16:1, 18:0, 18:1 18:1e, 18:2, 18:3, 20:0 20:1, 20:3, 20:4, and 22:5 was correlated with CRP (*p* = 0.003 to 0.049). The change in LPC 14:0, 15:0, 18:1 18:1e, 18:3, 20:0 20:1, 20:4, 26:0 was correlated with IL-8 (*p* = 0.004 to 0.048).

## 4. Discussion

This highly controlled diet intervention study sought to examine the impact of a DASH-style diet on changes in choline, choline metabolites, ceramides, and triglycerides in a cohort of obese adults aged 65–84 years. In response to the 12-week intervention, changes in plasma choline, DMG, total PC, total LPC, TMAO, total ceramide, and triglycerides resulted. Furthermore, the study diet influenced changes in individual LPC, sphingomyelin, and ceramide species.

### 4.1. In Response to the Dietary Choline and Betaine Provided by the DASH Diet, Plasma Choline Decreased and in Males Plasma Betaine Increased

The study diet provided an average of ~300 mg of dietary choline per day, which was delivered by food only, supplemental choline was not given. The amount of choline supplied by the study diet was below the adequate intake (AI) for adults which is 550 mg per day for males and 425 mg for females [48]. Males in the 3 oz group received ~47% of the choline AI, whereas males in the 6 oz group received ~61% of the choline AI. Females in the 3 oz group received ~60% of the choline AI and females in the 6 oz group received ~79% of the choline AI. Data from the 2013–2014 National Health and Nutrition Examination Survey (NHANES) showed that the average daily choline intake in adults from food and beverages is 402 mg in males and 278 mg in females [49]. Cross-sectional studies in adults from Canada and Tehran using food frequency questionnaires (FFQs) report an average dietary choline intake of 226 mg and 313 mg, respectively, with males consuming 372 mg and females 292 mg [50,51]. For the present study, the primary food sources of dietary choline were beef, eggs, and low-fat milk which are food items known to be high in choline [45]. For this study, beef and low-fat milk were provided every day during the 12-week intervention. Given that the study diet was within the DASH guidelines for meat intake and provided choline below the AI, individuals adhering to the DASH diet may need to increase intakes of choline-rich foods or consider a choline supplement to meet the recommendation. However, choline from food is more bioavailable compared to supplements [52].

In response to the study diet, plasma choline decreased by 9.6% from baseline to study end (*p* = 0.012; Table 4). Although there were no differences in plasma choline between the 3 oz and 6 oz beef intake groups at baseline or week 12 (Table 2), males had higher plasma choline compared to females (*p* = 0.042; Table 3) at baseline. The higher plasma choline in males may be an indication of a higher dietary choline intake at baseline. Plasma choline concentrations reflect dietary choline intake and males consume more choline than females [49,50,53,54,55]. In a 2-week randomized controlled crossover trial in individuals aged 17–70 years with a BMI range of 20–30 kg/m^2^, participants consumed 97%, 47%, and 25% of the choline AI [55]. In this study, plasma choline decreased from high choline intake to low choline intake. Similarly, the present study reports a decline in plasma choline over the 12-week intervention period in adults aged 65–84 years with a BMI of 32 kg/m^2^. Interestingly, in response to the study diet, the change in plasma choline was in association with %body fat (Table S1). Previously, we reported a decrease in %body fat and absolute fat mass in response to the diet intervention [38]. Numerous human studies show that dietary choline and plasma choline are associated with reductions in body fat and improved body composition [50,56,57,58]. Indeed, the body composition profile of this cohort of obese older adults improved in response to the study diet [35]. The association between choline and body fat in the present study may be due to the role of choline in hepatic fat removal and fatty acid oxidation. However, the amount of dietary choline for specific populations, such as obese older adults, that leads to an optimal reduction in body fat and improved body composition remains unknown and thus requires more investigation.

Estimated dietary betaine provided by the study diet had an average of 31.5 mg provided by food only, supplemental betaine was not given. The amount of betaine in the study diet was below dietary intakes reported by other studies. A 2017 systematic review and meta-analysis of six prospective cohort studies using FFQs reported a dietary betaine intake range of 41–478 mg in adults [59]. Large cross-sectional studies report an average betaine intake of 78 mg and 110 mg with males consuming more betaine compared to females [50,51]. Betaine is obtained primarily from grains, cereal, beets, and spinach [13,60,61]. In the present study, the main food sources of betaine were beef, spinach, and low-fat milk. Betaine functions primarily as an osmolyte and methyl-group donor with beneficial effects on alcohol-induced and metabolic-associated liver diseases, insulin resistance, cancer, and overall well-being [62]. Betaine also preserves heart function, prevents pancreatic disease, and has a neuroprotective role. Given that there are no dietary recommendations for betaine and the amount of betaine required to improve health is undetermined, it is unknown whether amounts typically consumed by adults are optimal to achieve improved health and thus require further investigation.

In the present study, males had higher plasma betaine compared to females at week 12 (*p* = 0.040; Table 3). Gao et al. showed that compared to females, males had higher circulating betaine concentrations in association with lower body fat and higher lean body mass [56]. In the present study, the change in plasma betaine was in association with %body fat (Table S1). A large cross-sectional study of >3200 Canadian adults aged 42 years reported a dose-dependent decline in %body fat with increased betaine intake [50]. However, dietary betaine assessed by FFQs showed that obese adults (BMI ≥ 30 kg/m^2^) consumed less betaine compared to non-obese adults and thus had a lower reduction in body fat. A 2019 systematic review and meta-analysis of six randomized controlled trials reported the effectiveness of betaine supplements on reducing body fat and improving body composition in adults aged 18–60 years with betaine doses of 2.5–10 g per day [63]. Betaine has also been shown to increase muscle mass, improve insulin signaling, and stimulate IGF-1 secretion [64,65,66]. Indeed, we previously reported that in response to the study diet there was an increase in muscle strength with a reduction in muscle loss in association with an improved cardiometabolic profile [38,39,40]. Furthermore, we showed a 10% increase in IGF-1 in association with decreased waist circumference and improved muscle function [39]. Although these associations suggest that betaine may have a role in the interrelationships between body composition and cardiometabolic outcomes, more studies are needed in diverse populations to better understand the function of betaine in body composition outcomes and the dietary intake amount required to achieve beneficial health outcomes.

### 4.2. Plasma Dimethylglycine Decreased in Response to the DASH Diet, and Males Had Higher DMG Compared to Females

In the present study, the average estimated amount of dietary methionine was 1654 mg. In response to the study diet, plasma DMG decreased by 10% (*p* = 0.042; Table 4). Methionine showed no significant change. Differing plasma DMG and methionine concentrations are reported in response to various diet intervention studies. The inconsistent outcomes may be due to the variances in study design, target population, length of study, and choline and betaine dietary sources. For example, in a randomized, controlled crossover trial individuals aged 35–70 years with metabolic syndrome consumed 400 mg of choline per day through eggs or supplemental choline for 4 weeks [67]. In response, DMG increased in both the egg and supplement groups. Methionine, however, did not change. In a 12-week parallel arm randomized placebo-controlled betaine supplement trial in individuals 21–70 years of age with obesity and prediabetes, plasma DMG increased by 16.5-fold and methionine by 1.5-fold in the supplement group compared to placebo [68]. However, this study provided ~5000 mg of supplemental betaine which is 2.5 times the typical betaine intake. A 4-week parallel, placebo-controlled intervention provided wheat cereal and bread as betaine food sources to the habitual diets of adults aged 45–65 years with a BMI of ≥25 kg/m^2^ [69]. In response, both plasma DMG and methionine were higher in the cereal and bread group compared to the control. Given that DMG synthesis results from the remethylation of homocysteine to form methionine through betaine-homocysteine methyltransferase (BHMT), the decreased plasma DMG in the present study may reflect a reduction in BHMT activity due to the lower dietary choline and betaine provided by the study diet [70,71]. Furthermore, BHMT activity may be sensitive to food or supplemental sources of choline and/or betaine. More investigation is needed to delineate the dietary patterns that impact circulating DMG and methionine levels and the implications of plasma concentrations for human health.

At baseline, males had higher plasma DMG (*p* = 0.001) and methionine (*p* = 0.003) compared to females and at week 12 the higher plasma DMG (*p* < 0.001) in males remained (Table 3). A community-based study conducted by Van Parys et al. in western Norway found that both middle-aged and elderly males had higher plasma methionine compared to females with a choline intake of 260 mg per day assessed by FFQs [72]. Unlike the present study, there were no changes in plasma DMG reported between males and females. Circulating concentrations of DMG and methionine may be associated with differential effects on health outcomes in older adults. For example, a 6-month nutritional supplement intervention, providing 55 mg of choline, in adults aged 65–98 years living in senior residences reported that increasing concentrations of DMG and methionine were associated with improved lipid metabolism and protection against cardiometabolic dysregulation [73]. However, findings from a large population-based prospective study in adults 60+ years of age showed that plasma DMG is associated with an increased risk for colorectal cancer, whereas plasma methionine was associated with a reduced risk of colorectal cancer [74]. Furthermore, outcomes from the Baltimore Longitudinal Study of Aging showed that older adults with low muscle quality presented with higher levels of plasma methionine [75]. Although we previously reported reduced muscle loss and improved muscle strength and cardiometabolic health in response to the study diet [35,36], we observed no associations between plasma DMG or methionine and body composition or cardiometabolic outcomes. The mechanisms by which DMG and methionine play a causal or associative role in older adult health require further investigation. Furthermore, studies are needed to delineate the impact of diet on circulating concentrations of DMG and methionine.

### 4.3. Plasma Phosphatidylcholine Decreased in Response to the Dietary Phosphatidylcholine Provided by the DASH Diet

An estimated average of 81.5 mg of dietary phosphatidylcholine (PC) was provided by the study diet. The primary food sources of dietary PC were beef and eggs and in the present study beef was the main PC food item. Conflicting outcomes on metabolic health related to beef or dietary PC have been reported. A 28-day randomized crossover controlled feeding trial in overweight and obese adults aged 18–74 years with prediabetes and/or metabolic syndrome showed that beef within the Healthy US-Style Eating Pattern had no effects on cardiometabolic health [76]. Similarly, a 2023 systematic review and meta-analysis of 21 randomized controlled trials reported that red meat consumption does not impact risk factors for T2DM [77]. Conversely, a large observational study of >22,000 adults in the United States showed that a higher PC intake, assessed by FFQs, is associated with the risk of all-cause mortality and cardiovascular disease, specifically in those with diabetes [78]. Outcomes of this study, however, were not interpreted within the context of diet quality or dietary pattern. In the present study, we investigated beef within the DASH diet in obese older adults and we observed improvements in cardiometabolic health [39]. Health outcomes related to dietary PC and/or beef may need to be interpreted within the context of dietary patterns and more studies that determine whether causal relationships exist between dietary PC and risk of adverse health outcomes are needed.

In response to the 12-week diet intervention, plasma total PC decreased by 51% (*p* < 0.001; Table 4). Diet quality and/or dietary pattern may differentially influence plasma PC concentrations. For instance, a prospective cohort study in colorectal cancer patients showed that adherence to the World Cancer Research Fund dietary recommendations was associated with lower plasma PC, whereas higher intakes of a carnivore or Western dietary pattern were associated with higher plasma PC levels [79]. The change in plasma PC in the present study was in association with skeletal muscle mass (Table S1). In a 3-week low-calorie diet intervention study in obese adults, changes in specific PC species in muscle tissue were associated with improved insulin sensitivity [80]. Moreover, plasma PC species are shown to be protective against insulin resistance and diabetes risk in older adults [81]. Conversely, older adults with low muscle quality are reported to have lower plasma concentrations of PC [75], potentially due to reduced mitochondrial function leading to decreased muscle function [82]. Although we previously reported improved insulin sensitivity and muscle strength in response to the study diet [38,39], the impact of or implications for reduced plasma PC on metabolic health in older adults are unknown and thus require further investigation.

### 4.4. In Response to the DASH Diet Total Lysophosphatidylcholine Increased

The physiological functions and specific mechanisms by which lysophosphatidylcholine (LPC) acts have not been fully elucidated and human clinical lipidomic trials report conflicting outcomes [83]. At baseline, participants randomized to the 3 oz beef intake group had higher plasma total LPC compared to participants randomized to the 6 oz beef intake group (*p* = 0.05; Table 2). Furthermore, in response to the diet, total LPC increased by 281% (*p* < 0.001; Table 4). This heightened increase in LPC may be due to the enhanced activity of phospholipase A, the enzyme responsible for converting PC to LPC [84,85]. Both overproduction and reduced concentrations of LPC are reported to be associated with unfavorable cardiometabolic outcomes, including diabetes, hypertension, and cardiovascular disease [83,86,87]. Furthermore, decreased LPC has been observed with aging [88]. In the present study, we report that the 281% increase in plasma total LPC is not in association with unfavorable cardiometabolic health outcomes. Although targeting LPC as a therapeutic option for treating cardiovascular disease has been suggested [83], optimal plasma LPC levels that are associated with improved health have not been established and the mechanisms underlying LPC’s harmful, or beneficial effects are not well understood. Thus, more rigorous human lipidomic clinical trials delineating the role of LPC in health are required.

### 4.5. Trimethylamine N-Oxide Increased in Response to a Higher Beef Intake

Trimethylamine *N*-oxide (TMAO) is a small organic compound derived from the oxidation of trimethylamine (TMA). Choline and L-carnitine serve as dietary precursors for TMAO synthesis and are both abundant in beef. The study diet provided an average of ~105 mg of dietary L-carnitine per day. In the present study, participants randomized to the 6 oz beef group had higher plasma TMAO at week 12 compared to those in the 3 oz beef group (*p* = 0.033; Table 2). Furthermore, in response to the study diet, TMAO increased by 26.5% (*p* < 0.001; Table 4). Plasma TMAO levels are influenced by the synthesis of TMA via gut microbes, which is likely a reflection of choline and L-carnitine supplied by the beef in the study diet. Circulating concentrations of TMAO have drawn attention as a potential mediator or biomarker of diseases such as obesity, cardiovascular disease, diabetes, kidney disease, and cancer [88,89,90,91,92,93,94]. Various randomized controlled feeding trials and observational cross-sectional studies in which diet was assessed through FFQs or diet records report conflicting results on TMAO concentrations and associations with unfavorable disease outcomes in humans [95,96,97,98,99,100,101,102,103,104,105]. The conflicts can likely be attributed to differences in study designs and characteristics and metabolic profiles of the study population. Although in the present study we observed that the change in plasma TMAO was in association with insulin and HOMA-IR (Table S1), the cardiometabolic profile of this cohort of obese older adults improved in response to the study diet [39]. The increase in plasma TMAO in the 6 oz beef group compared to the 3 oz group is likely due to the 6 oz group eating more beef. The question of whether TMAO is a cause or effect of impaired cardiometabolic health remains unknown [106,107] and until more is learned, changes in plasma TMAO should be interpreted within the context of diet, metabolic state, and/or changes within the gut microbiome.

### 4.6. Total Ceramide Decreased in Response to the DASH Diet

The bioactive sphingolipid ceramide receives attention due to its effects on insulin signaling and glucose utilization. Indeed, circulating ceramide concentrations are elevated in obese adults with T2DM and correlate with the severity of insulin resistance [28,30,108]. Reductions in plasma ceramide have been demonstrated to coincide with reduced inflammation and improvements in insulin signaling [30]. Numerous ceramide-reducing studies have been conducted using the potent drug myriocin which inhibits de novo synthesis of ceramide in the liver [109]. Use of myriocin has been shown to resolve insulin resistance and atherosclerosis and prevent diabetes and heart failure [109]. The ability, however, of diet to lower circulating ceramide is unexplored. In the present study, we observed a 22.1% reduction in total plasma ceramide from baseline to week 6 in response to the DASH diet used in this controlled feeding trial (*p* < 0.001; Table 4). Furthermore, we previously observed decreased inflammation and improved insulin signaling [39]. Similarly, an 8-week feeding trial using the 2010 MyPlate Dietary Guidelines for Americans (DGA), counseling, and principles of behavior change demonstrated that circulating total ceramide decreased by week 5 in association with improved inflammatory status in adults aged 18–28 years with a BMI of 26 kg/m^2^ [32]. In this study, total ceramide decreased by 16% in the fruit–vegetable–low refined carbohydrate group and 48% in the fruit–vegetable–low fat group. Taken together, these outcomes suggest that the DASH and DGA dietary patterns have the potential to reduce circulating ceramide in association with an improved metabolic health profile in adults with overweight or obesity. Future diet interventions, however, need to assess the extent to which the ceramide-lowering ability of such diets prevents the development of metabolic disease in adults.

### 4.7. In Response to the DASH Diet Plasma Triglycerides Decreased and Males Had Higher Triglycerides Compared to Females

Studies show that the DASH diet significantly reduces plasma triglycerides in middle-aged overweight adults and individuals with metabolic syndrome [110,111]. However, a 2019 umbrella systematic review and meta-analysis found no significant impact of the DASH diet on triglyceride concentrations [112]. In the present study, triglycerides decreased by 18% in response to the study diet (*p* = 0.021; Table 4). Several studies report associations between choline, betaine, and circulating triglycerides in middle-aged and older adults [73,99,113]. The associations are likely due to the role that choline and betaine play in triglyceride levels. Choline is essential for the export of triglycerides into very-low-density lipoprotein molecules and betaine suppresses triglyceride synthesis and uptake [114,115,116,117,118]. Conversely, high doses of betaine (4–6 g/day) and PC supplementation have been shown to increase triglycerides in humans [119,120]. In the present study, relationships between choline, betaine, and triglycerides were not observed, thus the decrease in triglycerides may be due to the impact of the DASH diet per se and not necessarily choline or betaine. Furthermore, it has been reported that calorie restriction and weight loss of at least 5% improve circulating triglycerides [121,122]. Indeed, we previously reported that the diet studied in this controlled feeding trial was restricted in calories throughout the 12-week intervention period and the participants lost 6% of total body weight by study end [38]. Lastly, 6 of the 28 participants self-reported taking statin medications, which may also be a contributing factor to the reduced triglyceride levels observed in the present study.

At baseline, participants randomized to the 3 oz beef group had higher (*p* = 0.023) plasma triglycerides compared to participants randomized to the 6 oz beef intake group (Table 2). By week 12, participants consuming 6 oz of beef had higher (*p* = 0.003) triglycerides compared to individuals consuming 3 oz of beef (Table 2), which may be due to the 6 oz group eating more beef. Although both males and females had lower plasma triglycerides at study end, males had higher (*p* = 0.003) triglycerides compared to females at week 12 (Table 3). Previous reports of NHANES data show that males tend to have higher circulating triglycerides compared to females [123,124]. While triglyceride levels of ≥150 mg/dL are associated with negative cardiometabolic outcomes, results from the present study showed no such relationships, and thus the higher triglycerides are attributed to the consumption of beef within the study diet.

### 4.8. Lysophosphatidylcholine Species Increased in Association with Biomarkers of Inflammatory and Muscle Health

Lysophosphatidylcholines (LPCs) are bioactive lipids investigated in the development of atherosclerosis and inflammation [22]. Conflicting outcomes on plasma concentrations of LPC species have been reported, resulting in difficulty interpreting results. For example, LPC species have been reported to be reduced and elevated in obesity and T2DM [18,19,20,21,22]. However, LPC outcomes are generally not interpreted within the context of nutritional status or diet. Studies performed by Kus et al. and Barber et al. suggest that diet plays an important role in determining and altering the profile of plasma LPC species [18,125]. In the present study, plasma levels of 20 LPC species were measured and in response to the study diet all species increased (*p* < 0.001; Table 5). Of the LPC species measured, LPC 16:0 had the greatest response (Table 5). LPC 16:0 is one of most abundant LPC species in plasma and has been shown to be related to metabolic health outcomes. In a case–control study investigating LPC species in adults with hypertriglyceridemia, LPC 16:0 was elevated and found to be a predictor of fasting triglyceride levels [126]. Outcomes from a lifestyle intervention trial involving insulin-sensitive and insulin-resistant adults with non-alcoholic fatty liver (NAFL) showed that LPC 16:0 was higher in the insulin-sensitive group and may serve as a biomarker for insulin sensitivity in individuals with NAFL [127]. In the present study, we observed that the change in LPC 16:0 and 20:4 was in association myostatin (Table S1). Myostatin is a myokine produced in skeletal muscle where it inhibits muscle growth. We previously reported that in response to the study diet myostatin decreased by 17.6% in association with increased muscle mass and strength [40]. Interestingly, low plasma LPC 18:2 has been shown to predict declines in gait speed in older adults [128]. Gait speed is a measure of physical function and predicts disability and mortality in older adults. Considering that LPCs may serve as a protector for skeletal muscle against lipotoxicity [129], further investigation is required to delineate the roles that individual LPC species may play in muscle health, particularly in older adults.

LPCs are widely considered to be potent pro-inflammatory lipids, however, recent discoveries suggest that LPCs may possess anti-inflammatory properties as well [22]. In the present study, we observed that 10 of the 20 LPC species correlated with C-reactive protein (CRP) (Table S1). CRP is a biomarker of inflammation and insulin resistance and is associated with coronary artery disease and total mortality [130,131,132]. We previously reported that in response to the study diet CRP decreased by 11.3% [39]. Previous studies show that LPC levels are inversely correlated with plasma CRP and LPC has the ability to bind CRP, resulting in delayed progression of atherosclerosis [133,134]. In the present study, we also observed that 10 of the 20 LPC species correlated with interleukin 8 (IL-8) (Table S1). IL-8 is a chemokine associated with an increased risk for cardiovascular disease but also reported to have beneficial cardioprotective effects [135,136]. In response to the study diet, we previously reported a 38.8% increase in IL-8 in association with decreased total cholesterol, reduced LDL-C, and decreased glucose, indicating a cardioprotective role for IL-8 [39]. The increased IL-8 we reported may be due to LPC’s ability to induce and promote IL-8 synthesis in endothelial cells [137,138]. While these associations collectively are suggestive of an anti-inflammatory role for LPCs in response to the DASH diet, this area remains largely unexplored. More studies are required to elucidate the relationship between diet and individual LPC species and delineate the impact of these relationships on cardiometabolic health in humans.

### 4.9. Sphingomyelin Species Respond Differentially in Association with Body Composition and Cardiometabolic Outcomes

Estimated dietary sphingomyelin provided by the study had an average of 17.5 mg. Dietary sphingomyelins are shown to be beneficial in lipid metabolism, cholesterol regulation, and in the prevention and treatment of metabolic diseases [139,140,141]. In rodents, dietary sphingomyelin from eggs lowered fat mass and had the potential to prevent atherosclerosis [142]. However, endogenous sphingomyelin species have been implicated in the etiology and pathogenesis of obesity, diabetes, atherosclerosis, and adipose tissue dysfunction [143,144,145,146,147,148]. In the present study, five sphingomyelin species were measured in plasma and of the five, sphingomyelin 16:0, 18:0, and 18:1 increased (all *p* < 0.001) by 10.4%, 22.5%, and 24%, respectively (Table 5). In contrast, we observed that sphingomyelin 24:0 significantly decreased by 10%. The change in sphingomyelin 18:0 and 18:1 was not in association with anthropometric or cardiometabolic outcomes. The relationships between circulating sphingomyelin species and health-related outcomes in humans differ and mechanisms that may explain relationships are not fully understood. For example, epidemiological and prospective cohort studies show that higher plasma sphingomyelin 16:0 is associated with incidence of heart failure, atrial fibrillation, and death, whereas higher plasma sphingomyelin 24:0 is related to lower risks of heart failure, atrial fibrillation, and death [23,24,25]. In the present study, sphingomyelin 16:0 significantly increased by 10.4% (Table 5) but was not in association with body composition or cardiometabolic outcomes. However, it is interesting to note that the 10.4% decrease in sphingomyelin 24:0 was associated with reduced total cholesterol, LDL-C, and glucose and increased IL-8 (Table S1). Individual sphingomyelin species are involved in numerous biological actions and the changes in plasma concentrations and associations reported in this study and others may be due to collective biological activity across multiple pathways and cell types occurring simultaneously. Albeit, in the present study, the changes reported are in response to a diet intervention that contained dietary sphingomyelins, which is not considered in other studies. Given that dietary sphingomyelin is associated with improved metabolic health outcomes in humans and may have an impact on the profile of sphingomyelin species, future studies that investigate changes in sphingomyelin species in response to diet are needed to better elucidate the relationship between dietary sphingomyelin, endogenous sphingomyelin species, and metabolic health.

At baseline, females had higher plasma sphingomyelin compared to males (*p* = 0.05; Table 3). Although sphingomyelin levels remained higher in females at week 12, they were not statistically different from males (*p* = 0.743; Table 3). Higher plasma levels of sphingomyelins in females have been previously reported. A longitudinal cohort study of community-dwelling adults aged 55 years and older investigating cross-sectional relationships between sphingomyelins and lifestyle factors found that women had higher plasma sphingomyelin species compared to men [149,150]. Furthermore, women in the highest tertile for all sphingomyelin species (except SM 24:1) had a significantly reduced risk of Alzheimer’s disease (AD), whereas higher sphingomyelin levels among men were associated with an increased risk of AD [150]. This suggests that differing plasma levels of sphingomyelin species may have differential effects on health in males and females, particularly those in the older adult population, which warrants further investigation.

### 4.10. In Response to the DASH Diet Ceramide Species Respond Differentially

The impact of diet on the profile of individual ceramide species is unexplored. In the present study, plasma concentrations of five ceramide species were measured. Although in response to the study diet total ceramide decreased (Table 4), there was a differential impact of the diet on individual ceramide species. In response to the study diet, ceramide 22:0 and 24:0 decreased by 26.2% (*p* < 0.001) and 11.2% (*p* = 0.002), respectively (Table 5). However, ceramide 24:1 increased by 37.5% (*p* = 0.054; Table 5). Mathews et al., however, reported that, in response to an 8-week diet intervention based upon the 2010 MyPlate DGA, ceramide 22:0, 24:0, and 24:1 decreased with an association between ceramide 22:0 and pro-inflammatory cytokines [32]. In the present study, the change in ceramide 22:0 was in association with total cholesterol (Table S1). The decreased ceramide 24:0 observed in the present study and by Mathews et al. may be viewed as favorable considering ceramide 24:0 is reported to be an antagonist of insulin action described as inhibiting glucose transport in skeletal muscle and negatively impacting insulin-stimulated glucose disposal [151,152]. However, high concentrations of ceramide 24:0 are associated with a reduced risk of atrial fibrillation and heart failure [24,25]. Although there is a consensus that ceramide may serve as a biomarker of metabolic disease in humans, the impact of diet on determining and altering the profile of ceramide species in various human populations and the relationship to human health warrants further investigation. Furthermore, clinical trials are needed to investigate the influence of diet on the divergent responses of ceramide species in human health.

### 4.11. Limitations

Limitations of the present study include: (i) although two different amounts of beef were compared, this study lacked a non-beef comparison group; (ii) all participants self-identified as white American which is representative of the main racial group in South Dakota; (iii) no participants required support for daily living activities and lived independently in their own homes; (iv) South Dakota is a rural state. These limitations should be considered when generalizing outcomes from this study to diverse populations of older adults including those with differing demographic and ethnic backgrounds and living environments.

## 5. Conclusions

Results from this controlled feeding diet intervention trial in obese older adults show that in response to the DASH diet that provided dietary choline below the AI, plasma choline, PC, and total ceramide decreased, while plasma total LPC and TMAO increased. Outcomes also demonstrated distinct patterns of change in plasma LPC, sphingomyelin, and ceramide species, suggesting that the DASH diet may influence the determination and modification of individual lipid species. Given that the effect of diet on changes in specific phospholipid and sphingolipid molecules in relation to metabolic health outcomes in humans remains largely unexplored, additional studies are required to determine the long-term clinical effects and relevance of these relationships, particularly in vulnerable populations such as older adults.

## Figures and Tables

**Table 1:** Estimated dietary choline, betaine, choline forms, L-carnitine, and methionine provided by the study diet.

	Estimated Dietary Amount (mg/d)
* Total Choline	
Diet + 3 oz beef	257
Diet + 6 oz beef	336
* Betaine	
Diet + 3 oz beef	28
Diet + 6 oz beef	35
^+^ Glycerophosphocholine	
Diet + 3 oz beef	50
Diet + 6 oz beef	53
^+^ Phosphocholine	
Diet + 3 oz beef	13
Diet + 6 oz beef	13
^+^ Phosphatidylcholine	
Diet + 3 oz beef	78
Diet + 6 oz beef	85
^+^ Sphingomyelin	
Diet + 3 oz beef	14
Diet + 6 oz beef	21
^#^ L-Carnitine	
3 oz beef only	70
6 oz beef only	140
^^^ Methionine	
Diet + 3 oz beef	1350
Diet + 6 oz beef	1957

* Estimated using 2022 USDA FoodCentral database [45]. ^+^ Estimated using 2008 USDA Database for the Choline Content of Common Foods [46]. ^^^ Estimated using Nutritionist Pro^®^ Software. ^#^ Estimated using NIH Office of Dietary Supplements Carnitine Fact Sheet for Health Professionals [47].

**Table 2:** Baseline and week 12 variable characteristics separated by beef group.

	Baseline			Week 12				
Variables	3 oz Beef Group (n = 15)	6 oz Beef Group (n = 13)	* *p*-Value	3 oz Beef Group (n = 15)	% Change from Baseline	6 oz Beef Group (n = 15)	% Change from Baseline	** *p*-Value
Choline (nM/mL)	11.9 (2.8)	11.4 (2.4)	0.643	11.6 (2.9)	−3.4 (19.6)	11.5 (2.3)	−10.6 (22.1)	0.388
Betaine (nM/mL)	37.9 (12.2)	35.1 (6.7)	0.493	37.3 (11.9)	3.4 (24.0)	35.0 (6.5)	−1.7 (30.7)	0.646
Methionine (μM/mL)	34.85 (7.14)	32.09 (5.10)	0.268	32.69 (4.18)	−3.57 (22.75)	32.67 (6.78)	2.60 (18.36)	0.973
DMG (μM/mL)	2.81 (0.70)	2.42 (0.73)	0.504	2.52 (0.84)	−8.32 (17.83)	2.15 (0.71)	−8.98 (21.44)	0.223
Total PC (μg/mL)	1448.7 (530.6)	1315.0 (550.3)	0.526	643.2 (145.1)	−55.6 (14.6)	721.9 (156.0)	−45.1 (23.6)	0.196
Total LPC (μg/mL)	30.7 (3.4)	27.9 (3.8)	0.050	110.0 (7.6)	258.3 (33.6)	114.3 (7.6)	309.7 (64.1)	0.171
Sphingomeylin (μM/mL)	132.7 (19.0)	139.0 (23.4)	0.503	140.4 (15.87	8.6 (16.4)	142.9 (18.4)	5.9 (21.4)	0.743
TMAO (nM/mL)	4.3 (1.6)	4.5 (3.5)	0.980	4.6 (2.1)	12.0 (44.1)	5.2 (3.4)	61.7 (65.8)	0.033
L-Carnitine (nM/mL)	54.1 (48.5)	49.2 (37.1)	0.631	62.2 (26.5)	25.3 (48.7)	61.4 (29.9)	18.1 (62.3)	0.745
Total Ceramide (μM/mL)	10.2 (1.0)	10.2 (2.5)	0.944	9.1 (1.8)	−8.6 (16.6)	9.1 (2.7)	−8.4 (24.2)	0.962
Triglycerides (mg/dL)	224 (82)	155 (70)	0.023	149 (74)	−28.5 (35.5)	167 (65)	14.3 (32.8)	0.003

* Baseline data are presented as means and standard deviations except for TMAO and L-carnitine which are reported as median (interquartile range). Independent samples *t*-tests or the Mann–Whitney U test (for non-normal data) was performed to determine group differences in baseline characteristics by beef intake group. DMG, dimethylglycine; PC, phosphatidylcholine; LPC, lysophosphatidylcholine; TMAO, trimethylamine N-oxide. ** Week 12 data are presented as means and standard deviations. Independent samples *t*-tests were performed to determine group differences in week 12 characteristics by beef intake group.

**Table 3:** Baseline and week 12 variable characteristics separated by sex.

Baseline					Week 12				
Variables	Total (n = 28)	Female (n = 17)	Male (n = 11)	* *p*-Value	Female (n = 17)	% Change from Baseline	Male (n = 11)	% Change from Baseline	** *p*-Value
Choline (nM/mL)	11.5 (2.6)	10.7 (2.6)	12.7 (2.3)	0.042	9.8 (2.2)	−5.6 (20.9)	11.3 (1.9)	−8.9 (21.5)	0.080
Betaine (nM/mL)	36.1 (9.5)	33.3 (7.4)	40.0 (11.0)	0.097	31.5 (8.5)	−2.2 (27.1)	41.4 (12.8)	5.0 (27.9)	0.040
Methionine (μM/mL)	33.47 (6.24)	30.51 (4.33)	37.51 (6.33)	0.003	31.75 (5.85)	4.6 (22.6)	34.12 (4.60)	−7.5 (15.8)	0.277
DMG (μM/mL)	2.61 (0.73)	2.23 (0.57)	3.13 (0.60)	0.001	1.95 (0.43)	−11.5 (17.1)	2.96 (0.85)	−4.8 (22.3)	<0.001
Total PC (μg/mL)	1384.3 (534.0)	1449.4 (634.3)	1289.7 (348.4)	0.456	671.5 (175.8)	−51.5 (24.6)	692.4 (113.2)	−46.3 (13.1)	0.732
Total LPC (μg/mL)	29.4 (3.8)	28.9 (4.1)	30.0 (3.4)	0.471	110.6 (8.9)	283.8 (61.5)	114.0 (5.2)	289.4 (45.9)	0.231
Sphingomeylin (μM/mL)	134.8 (21.0)	141.5 (22.5)	124.7 (12.9)	0.050	143.1 (18.7	3.9 (18.3)	139.3 (13.8)	13.0 (19.1)	0.743
TMAO (nM/mL)	4.3 (2.6)	4.3 (3.0)	4.1 (1.7)	0.610	6.6 (3.9)	40.9 (63.2)	5.5 (2.7)	31.0 (58.8)	0.381
L-Carnitine (nM/mL)	52.3 (37.4)	50.4 (44.9)	54.1 (46.7)	0.241	64.3 (28.2)	19.5 (60.5)	82.3 (35.9)	26.5 (44.2)	0.175
Total Ceramide (μM/mL)	10.2 (1.9)	10.1 (2.1)	10.4 (1.6)	0.700	8.5 (2.0)	−16.9 (20.9)	10.1 (2.2)	−2.9 (9.7)	0.082
Triglycerides (mg/dL)	192 (83)	185 (76)	203.2 (95.2)	0.576	148 (69)	−9.3 (45.2)	170 (71)	−7.6 (32.5)	0.003

* Baseline data are presented as means and standard deviations except for TMAO and L-carnitine which are reported as median (interquartile range). Independent samples *t*-tests or the Mann–Whitney U test (for non-normal data) was performed to determine group differences in baseline characteristics by sex. DMG, dimethylglycine; PC, phosphatidylcholine; LPC, lysophosphatidylcholine; TMAO, trimethylamine N-oxide. ** Week 12 data are presented as means and standard deviations. Independent samples *t*-tests were performed to determine group differences in week 12 characteristics by sex.

**Table 4:** Choline, choline metabolites, ceramide, and triglycerides in response to the 12-week diet intervention.

Weeks of Intervention				
Variables	0	6	12	*p*-Value
Choline (nM/mL)	11.5 (2.6)	10.6 (2.1)	10.4 (2.2) *	0.012
Betaine (nM/mL)	32.9 (14.7)	34.9 (9.4)	33.3 (19.5)	0.141
Methionine (μM/mL)	32.1 (8.7)	31.5 (6.1)	31.5 (7.4)	0.289
DMG (μM/mL)	2.62 (1.24)	2.26 (1.14)	2.27 (1.02) *	0.042
Total PC (μg/mL)	1384.3 (534.0)	1407.0 (189.3)	679.5 (152.5) †	<0.001
Total LPC (μg/mL)	29.4 (3.8)	75.6 (14.5) †	112.0 (7.7) †	<0.001
Sphingomyelin (μM/mL)	134.8 (21.0)	140.1 (13.6)	142.3 (17.7)	0.275
TMAO (nM/mL)	4.9 (2.8)	6.1 (2.9)	6.2 (3.5) *	<0.001
L-Carnitine (nM/mL)	52.5 (48.8)	58.5 (42.7)	68.4 (35.2)	0.840
Total Ceramide (μM/mL)	10.2 (1.9)	8.1 (1.8) †	9.3 (2.3) *	<0.001
Triglycerides (mg/dL)	192.0 (83.1)	162.6 (67.0) *	157.0 (69.3) *	0.021

Data are presented as means and standard deviations, except for betaine, methionine, DMG, and L-carnitine which are reported as median (interquartile range). For normally distributed data, a mixed design ANOVA was used to determine changes in the primary outcome variables across the intervention. For betaine, methionine, DMG, and L-carnitine, the Friedman’s test was used to determine changes from baseline to week 12. DMG, dimethylglycine; PC, phosphatidylcholine; LPC, lysophosphatidylcholine; TMAO, trimethylamine N-oxide. † *p* < 0.001 versus week 0; * *p* < 0.05 versus week 0.

**Table 5:** Changes in LPC, sphingomyelin, and ceramide species in response to the diet intervention.

Variable		Baseline			Week 12		Weeks of Intervention			
LPC (µg/mL)	3 oz	6 oz	*p*-Value	3 oz	6 oz	*p*-Value	Week 0	Week 6	Week 12	*p*-Value
14:0	0.07 (0.02)	0.07 (0.02)	0.971	0.17 (0.02)	0.17 (0.02)	0.086	0.07 (0.04)	0.12 (0.09) †	0.17 (0.03) †	<0.01
15:0	0.07 (0.01)	0.08 (0.01)	0.067	0.60 (0.08)	0.61 (0.08)	0.344	0.08 (0.05)	0.33 (0.16) †	0.59 (0.11) †	<0.01
16:0	7.41 (2.05)	7.69 (2.03)	0.725	54.98 (6.99)	54.79 (7.20)	0.175	7.77 (3.09)	35.34 (15.65) †	52.29 (7.57) †	<0.01
16:0e	0.11 (0.01)	0.09 (0.03)	0.091	0.15 (0.03)	0.17 (0.03)	0.230	0.11 (0.02)	0.24 (0.04) †	0.16 (0.02) †	<0.01
16:1	0.37 (0.10)	0.36 (0.09)	0.695	1.03 (0.38)	1.26 (0.38)	0.140	0.35 (0.16)	0.83 (0.04) †	1.26 (0.64) †	<0.01
16:1e	0.05 (0.01)	0.04 (0.01)	0.992	0.53 (0.37)	0.51 (0.07)	0.037	0.05 (0.01)	0.34 (0.13) †	0.50 (0.08) †	<0.01
17:0	0.48 (0.18)	0.50 (0.15)	0.759	1.43 (0.17)	1.48 (0.14)	0.610	0.49 (0.31)	0.93 (0.42) †	1.47 (0.22) †	<0.01
18:0	8.48 (1.35)	7.22 (1.68)	0.042	15.77 (2.23)	16.93 (2.20)	0.202	8.02 (2.47)	12.54 (4.58) †	16.64 (2.24) †	<0.01
18:1	4.40 (0.74)	3.63 (0.88)	0.024	9.15 (2.46)	10.74 (2.56)	0.606	4.16 (1.02)	8.80 (4.64) †	9.86 (3.70) †	<0.01
18:1e	0.08 (0.01)	0.08 (0.01)	0.587	0.18 (0.05)	0.18 (0.05)	0.738	0.08 (0.01)	0.19 (0.03) †	0.17 (0.08) †	<0.01
18:2	6.26 (0.99)	5.20 (1.30)	0.027	16.24 (2.69)	16.99 (2.18)	0.111	5.97 (1.74)	10.03 (6.38) †	16.54 (2.41) †	<0.01
18:3	0.88 (0.17)	0.78 (0.16)	0.134	2.55 (0.39)	2.79 (0.37)	0.436	0.83 (0.21)	1.34 (0.99) †	2.77 (0.64) †	<0.01
20:0	0.04 (0.01)	0.04 (0.01)	0.070	0.09 (0.01)	0.10 (0.01)	0.202	0.04 (0.01)	0.06 (0.04) †	0.09 (0.02) †	<0.01
20:1	0.05 (0.01)	0.04 (0.01)	0.950	0.17 (0.03)	0.19 (0.03)	0.317	0.04 (0.02)	0.14 (0.08) †	0.18 (0.05) †	<0.01
20:3	0.52 (0.15)	0.49 (0.15)	0.711	1.64 (0.35)	1.85 (0.36)	0.146	0.49 (0.24)	0.99 (0.60) †	1.80 (0.53) †	<0.01
20:4	0.84 (0.12)	0.92 (0.24)	0.323	2.82 (0.60)	3.08 (0.68)	0.647	0.91 (0.31)	4.93 (1.59) †	2.79 (1.27) †	<0.01
20:5	0.18 (0.03)	0.16 (0.03)	0.205	1.38 (0.18)	1.35 (0.19)	0.094	0.17 (0.05)	0.43 (0.29) †	1.32 (0.27) †	<0.01
22:5	0.05 (0.01)	0.05 (0.01)	0.104	0.16 (0.03)	0.18 (0.03)	0.201	0.05 (0.02)	0.18 (0.11) †	0.17 (0.04) †	<0.01
22:6	0.34 (0.13)	0.44 (0.12)	0.070	0.85 (0.08)	0.80 (0.05)	0.654	0.39 (0.23)	0.56 (0.23) †	0.82 (0.06) †	<0.01
26:0	0.03 (0.01)	0.04 (0.01)	0.250	0.07 (0.01)	0.08 (0.01)	0.033	0.03 (0.01)	0.04 (0.01) †	0.08 (0.01) †	<0.01
Sphingomyelin (μM/mL)										
16:0	44.8 (9.3)	46.1 (10.3)	0.744	48.4 (7.9)	51.7 (8.0)	0.312	45.3 (9.6)	49.4 (6.2)	50.0 (8.0) *	<0.001
18:0	10.6 (3.5)	11.7 (4.9)	0.507	13.1 (4.6)	14.3 (5.3)	0.552	11.1 (4.4)	12.9 (5.4)	13.6 (4.9) *	<0.001
18:1	4.5 (1.2)	5.3 (1.9)	0.238	5.7 (1.4)	6.5 (2.2)	0.284	4.8 (4.6)	6.1 (1.8) †	6.0 (1.9) †	<0.001
24:0	33.9 (10.9)	35.6 (8.0)	0.652	32.1 (6.9)	28.9 (5.6)	0.209	34.1 (9.9)	29.9 (5.7) †	30.8 (5.8) †	<0.001
24:1	38.9 (5.6)	39.8 (7.2)	0.744	41.1 (6.2)	41.5 (7.5)	0.897	39.5 (6.1)	41.3 (11.1)	40.1 (6.9)	0.384
Ceramide (µM/mL)										
C12:0	0.24 (0.11)	0.34 (0.12)	0.444	0.28 (0.08)	0.29 (0.10)	0.529	0.31 (0.11)	0.28 (0.08)	0.28 (0.10)	0.180
C16:0	0.29 (0.23)	0.58 (1.15)	0.479	0.55 (0.33)	0.65 (0.60)	0.703	0.35 (0.51)	0.39 (0.39)	0.56 (0.41)	0.368
C22:0	1.02 (0.78)	1.31 (0.74)	0.470	0.89 (0.61)	0.89 (1.06)	0.892	1.22 (0.73)	0.60 (0.49) †	0.90 (0.77) †*	< 0.001
C24:0	7.83 (1.02)	7.60 (1.46)	0.479	7.56 (1.87)	6.71 (3.24)	0.703	7.60 (1.30)	6.16 (2.28) †	6.75 (2.78) †*	0.002
C24:1	0.48 (0.27)	0.56 (0.30)	0.270	0.54 (0.68)	0.78 (0.45)	0.320	0.56 (0.29)	0.75 (0.55)	0.77 (0.52)	0.054

Data are presented as means and standard deviations, except for LPC (weeks of intervention) and ceramide species which are reported as median (interquartile range). For normally distributed data, independent samples *t*-tests were performed to determine differences at baseline and week 12 by beef intake group, and mixed design ANOVA was used to determine changes from baseline to week 12. For ceramide species, the Mann–Whitney U test was performed to determine differences at baseline and week 12 by beef intake group. The Friedman’s test was used to determine changes from baseline to week 12 for LPC and ceramide species. LPC, lysophosphatidylcholine. † *p* < 0.01 versus week 0; * *p* < 0.05 versus week 6.

## Data Availability

Data presented are contained within this article and Supplementary Materials.

## Supplementary Materials

The following supporting information can be downloaded at: https://www.mdpi.com/article/10.3390/nu15173687/s1, Table S1: Correlations between plasma choline, betaine, PC, L-carnitine, TMAO, sphingomyelins, ceramides, and LPCs and body composition, cardiometabolic, and inflammatory markers.

## Abbreviations

AD	Alzheimer’s Disease
BMI	Body Mass Index
CVD	Cardiovascular Disease
DASH	Dietary Approaches to Stop Hypertension
DGA	Dietary Guidelines for Americans
DMG	Dimethylglycine
FFQ	Food Frequency Questionnaire
GPC	Glycerophosphotidylcholine
LDL-C	Low-Density Lipoprotein Cholesterol
LPC	Lysophosphatidylcholine
NHANES	National Health and Nutrition Examination Survey
PC	Phosphatidylcholine
SMM	Skeletal Muscle Mass
TMAO	Trimethylamine N-Oxide
T2DM	Type-2 Diabetes Mellitus
US	United States
USDA	United States Department of Agriculture
