# Supplementary material for: Changes in Choline Metabolites and Ceramides in Response to a DASH-Style Diet in Older Adults
Source: Nutrients. 2023 Aug 23;15(17):3687. doi: 10.3390/nu15173687 (PMC10489641; doi:10.3390/nu15173687)
Supplement: Supplementary file 1 [file nutrients-15-03687-s001.zip › nutrients-2533265-supplementary.pdf]

Table S1: Correlations between plasma choline, betaine, PC, L-carnitine, TMAO, sphingomyelins, ceramides, and LPCs with body composition, cardiometabolic, and inflammatory markers

	Anthropometric, muscle, cardiometabolic, and inflammatory markers													
**Variable**	**BW**	**BMI**	**SMM**	**% BF**	**Grip strength**	**Myostatin**	**TC**	**LDL-C**	**Glucose**	**TG**	**Insulin**	**HOMA-IR**	**CRP**	**IL-8**
Choline				-0.44										
Betaine				-0.49										
PC			-0.42											
L-Carnitine									-0.49					0.47
TMAO											0.51	0.50		
Sphingomyelins														
24:0							-0.42	-0.49	-0.63					0.74
24:1	-0.61	-0.61	-0.47		0.51									
Ceramides														
C22:0							0.44							
LPCs														
14:0														0.44
15:0														-0.56
16:0						0.48								
16:1													-0.43	
16:1e									0.52					
18:0									0.42				-0.45	
18:1										0.42			-0.54	0.41
18:1e													-0.44	0.53
18:2				0.41									-0.39	
18:3													-0.55	0.40
20:0													-0.57	0.55
20:1										0.55			-0.39	0.47
20:3													-0.44	0.37
20:4	0.39	-0.39				-0.55	-0.43	-0.40						0.55
22:5													-0.52	
26:0														0.53

Relations were performed by Pearson’s correlation coefficient. PC, phosphatidylcholine; TMAO, trimethylamine N-oxide. DMG, dimethylglycine; LPC, lysophosphatidylcholine; BW, body weight; BMI, body mass index; SMM, skeletal muscle mass; %BF, percent body fat; TC, total cholesterol; LDL-C, low-density lipoprotein cholesterol; TG, triglyceride; HOMA-IR, homeostatic model assessment for insulin resistance; CRP, C-Reactive Protein; IL-8, Interleukin-8.
